# Editorial: eHealth and personalized medicine in mental health and neurodevelopmental disorders: digital innovation for diagnosis, care, and clinical management

**DOI:** 10.3389/fpsyt.2026.1932293

**Published:** 2026-07-21

**Authors:** Francesco Monaco, Annarita Vignapiano, Luca Steardo

**Affiliations:** 1 Department of Mental Health, Azienda Sanitaria Locale (ASL) Salerno, Salerno, Italy; 2 European Biomedical Research Institute of Salerno (EBRIS), Salerno, Italy

**Keywords:** artificial intelligence, digital phenotyping, digital therapeutics, eHealth, mental health screening, neurodevelopmental disorders, precision psychiatry, wearable devices

## Introduction

Digital transformation is reshaping every stage of psychiatric care, from diagnosis to long-term monitoring and personalized intervention. Mental health and neurodevelopmental disorders (NDDs) are among the most heterogeneous categories in medicine, resisting one-size-fits-all protocols and motivating a shift toward personalized and precision approaches. Over the past decade, eHealth technologies, including artificial intelligence (AI), machine learning, wearable sensors, mobile applications, virtual reality, and remote monitoring, have moved from proof-of-concept to clinically oriented tools that provide objective, continuous, and individualized data where clinical observation alone is limited by recall bias, low sampling frequency, and rater subjectivity. AI is increasingly becoming the enabling framework that integrates multimodal data from wearables, smartphones, electronic health records, and behavioral monitoring into clinically actionable knowledge. This Research Topic gathered 29 contributions across four lines of work: objective assessment of neurodevelopmental conditions, passive physiological monitoring, digital therapeutics and care-pathway implementation, and screening or predictive tools for risk and outcome.

## Objective, technology-assisted assessment of neurodevelopmental disorders

A substantial cluster of contributions addressed ADHD through instrumented, rater-independent measurement. Kayış et al. contributed three related studies using point-of-view (POV) cameras and machine learning: two studies focused on objective hyperactivity assessment in ADHD (including a preschool-specific paradigm with MediaPipe), and a third study extended the same approach to multimodal depression detection, demonstrating that passive behavioral capture generalizes across diagnostic targets. Meng et al. presented a complementary machine learning feature selection approach for ADHD prediction, while Schofield et al. provided a scoping review of digital health technology for adults with ADHD, a population underserved by tools designed for children. Kraut et al. conducted a randomized controlled trial to test whether the format of the Adult ADHD Self-Report Scale affects screen-positive rates, a reminder that digitizing an instrument does not remain neutral to its performance. Ophir et al. used AI-assisted text analysis to trace how the ADHD construct has shifted across six DSM editions. Beyond ADHD, Tiwari et al. presented “Akshar Mitra,” a multimodal framework for early dyslexia detection, and Di Vincenzo et al. contributed with a mini review on digital health in Tourette syndrome. Collectively, these contributions illustrate a shift from subjective symptom reporting to objective, technology-enabled phenotyping, an approach intended to complement rather than replace clinical judgment.

## Passive physiological and behavioral monitoring

A second cluster of studies moved from clinic-based assessment to continuous, out-of-clinic data capture. Matsuda and Shinohara reported a case study on the feasibility of one-month, home-based heart rate variability (HRV) monitoring in ASD using smart clothing; Eto et al. presented a case report on the use of wearable-derived HRV and sleep data to predict mood episodes in bipolar disorder. Lee et al. used deep learning to detect stress in RR-interval data across major depressive disorder, panic disorder, and healthy controls, and Kim et al. used simulated virtual reality experiences to predict early treatment response in panic disorder. Together, these studies point toward continuous, ecologically valid observation as a complement to episodic clinical evaluation.

## Digital therapeutics and care-pathway implementation

A third, more intervention-oriented cluster of articles spanned from individual platforms to health system deployment. Del Vecchio et al. described “My Cosmos,” a gamified transdiagnostic digital CBT platform, and Engelkamp et al. presented a protocol for therapist-guided digital CBT for avoidant/restrictive food intake disorder (ARFID) in children and their parents. On a larger scale, Orsolini et al. reported their findings from DIGIT-PSY, a multicenter Italian study of digital mental health practices; Abi Ramia et al. evaluated the real-world scaling up of Step-by-Step in Lebanon; Rombos et al. described the implementation of measurement-based care within Ontario’s Extensive Needs Service; and Ye, Zhang, and Zhou synthesized evidence on mobile health interventions for schizophrenia in a meta-analysis. Wang et al. extended the family-participatory, cloud-based follow-up model to patients with chronic, difficult-to-heal wounds, evaluating psychological well-being alongside physical outcomes. This is a boundary case for this Research Topic, but it was included here for its psychological well-being endpoint. Taken together, these contributions suggest that digital technologies are moving from standalone experiments toward integration in routine care.

## Screening, prediction, and outcome measurement

A fourth cluster of contributions addressed how digital tools can identify risk factors or track outcomes at scale. Jeon et al. validated smartphone-based neuropsychological tasks for screening subclinical depression and anxiety; Fu et al. used machine learning and SHAP analysis to identify predictors of oral frailty in hospitalized patients with schizophrenia; Hasan et al. and Quiram et al. each contributed reviews: one on machine-learning methods for mental health state detection and the other on barriers and tools in remote mental health screening. Grant et al. conducted a systematic review of non-verbal behaviors as predictors of treatment response in depression and schizophrenia, while Băcilă et al. built a predictive model of risk factors for involuntary psychiatric admission before and during COVID-19. El Alaoui et al. examined guideline adherence in depression outcome measurement within Swedish psychiatric outpatient care, connecting digital measurement infrastructure to actual clinical practice. Overall, these studies point toward a more proactive, data-informed model of risk identification and treatment monitoring.

## Information ecosystems

Two contributions stepped outside the clinical-tool paradigm to examine the informational environment patients encounter. Alpua and Yoldaş assessed the reliability of YouTube content on functional neurological disorder, while Alimour and Alrabeei proposed a reconceptualization of emerging, digitally mediated psychopathologies under conditions of information saturation. Both studies are relevant to eHealth strategies, since the tools described elsewhere in this topic do not operate in an informational vacuum. These contributions are a reminder that digital innovation extends beyond clinical tools to the informational environment that patients navigate on their own.

## Synthesis and open questions

Taken together, these contributions confirm a field that is moving unevenly across the innovation pipeline. Instrumented, objective assessment is maturing fastest, since these approaches can be validated against existing scales without long-term outcome data. Digital therapeutics and system-level implementation are in the early stages, dominated by protocols, feasibility studies, and single-site evaluations rather than multisite controlled trials. Several contributions are case reports or small feasibility studies; cross-study comparability is limited by heterogeneous outcome measures and platforms; and few address data governance, interoperability, or the equity implications of tools presupposing smartphone or wearable access. The future of digital psychiatry depends not only on new technologies but also on AI’s ability to integrate heterogeneous clinical, behavioral, and physiological data into transparent, reliable decision support systems. Precision psychiatry and eHealth will earn their place in routine care only to the extent that they are held to the same evidentiary standard as any other clinical intervention.

